
JOURNAL INFORMATION
==============================
NLM Title Abbreviation: Behav Neurol
Journal ID: Behav Neurol
NLM Journal ID: 8914585
Journal ID: BN

Behavioural Neurology

ISSN: 0953-4180
EISSN: 1875-8584
Publisher: Wiley

ARTICLE INFORMATION
==============================
PMCID: PMC5434354
PMID: 21422562
DOI: 10.3233/BEN-2010-0302
Article ID: 928680
Article version: 1
Subjects: Research Article

Acquired Prosopagnosia Is Not due to a General Impairment in Fine-Grained Recognition of Exemplars of a Visually Homogeneous Category

Busigny Thomas *
Rossion Bruno
Université Catholique de Louvain Louvain-la-Neuve Belgium
*Thomas Busigny: thomas.busigny@uclouvain.be
Print publication date: 2010
Electronic publication date: 2011 Mar 7
Volume: 23
Issue: 4
First page: 229
Last page: 231
Received 2011 Jan 31; Accepted 2011 Jan 31
Copyright: Copyright © 2010 Hindawi Publishing Corporation and the authors.
Copyright year: 2010
License: This is an open access article distributed under the Creative Commons Attribution License, which permits unrestricted use, distribution, and reproduction in any medium, provided the original work is properly cited.
License URL: https://creativecommons.org/licenses/by/3.0/

==============================
Behavioural Neurology 23 (2010) 229–231
DOI 10.3233/BEN-2010-0302
IOS Press

### Acquired prosopagnosia is not due to a general impairment in fine-grained recognition of exemplars of a visually homogeneous category

Thomas Busigny* and Bruno Rossion
*Université Catholique de Louvain, Louvain-la-Neuve, Belgium*

### 1. Introduction

The understanding of the nature of prosopagnosia – classically defined as a disorder of face recognition following brain damage – remains largely unclear and debated. One of the major debates concerns the question of whether prosopagnosia affects only the category of faces or whether it also affects some other categories. Regarding this second position, and according to a long-standing view, prosopagnosia could reflect a general difficulty for fine-grained discrimination in visually homogenous object categories, including faces [5–7]. Taking over this idea within an experimental context, Gauthier and colleagues [7] tested two cases of acquired prosopagnosia in a set of visual discrimination tasks. The two patients were described as showing steeper increases of error rates and correct RTs as the visual similarity between the distractor and the target increased. These observations were taken as evidence against the domain-specificity account of acquired prosopagnosia, and in favor of the view that the syndrome should be better characterized as an impairment in discriminating items at subordinate levels of categorization (i.e., visually similar), regardless of object category. However, even if this position has long been influential, very little empirical evidence supports this view (see [2]). On the one hand, the prosopagnosic patients tested both by Damasio et al. [5] and Gauthier et al. [7] all complained and presented with severe deficits at recognizing nonface objects at the basic level, that is, they suffered from a general visual agnosia syndrome to start with. Thus, they were certainly not the best cases of acquired prosopagnosia to test the alternative hypothesis to the domain-specificity account. On the other hand, the few studies that reported cases of prosopagnosia who do not complain of object recognition difficulties did not test the visual similarity account with objective and parametric manipulations of visual similarity.

In the present study, we tested the hypothesis that acquired prosopagnosia may be due, or be directly related, to a general difficulty at discriminating visually similar exemplars of a nonface category. We tested this hypothesis stringently with three brain-damaged prosopagnosic patients who have no difficulties at basic-level object recognition: PS [9], LR [1] and GG [3]. We report an experiment that tested prosopagnosic patients' discrimination of individual exemplars of cars and faces. The task was a two-alternative forced-choice matching in which the similarity of the distractor and the target item was increased parametrically (see methodology in Busigny et al., 2010 [2]). Each patient was compared to a group of gender- and age-matched controls.

For cars, PS, LR and GG showed normal performance and speed at each level of visual similarity. Fur-

*Address for corresponding: Thomas Busigny, Université Catholique de Louvain (UCL), Faculté de Psychologie et des Sciences de l'Education (PSP), Unité de Cognition et Développement (CODE), Place du Cardinal Mercier, 10, B-1348 Louvain-la-Neuve, Belgium. Tel.: +32 (0) 10 47 92 60; Fax: +32 (0) 10 47 37 74; E-mail: thomas.busigny@uclouvain.be.

ISSN 0953-4180/10/$27.50 © 2010 – IOS Press and the authors. All rights reserved

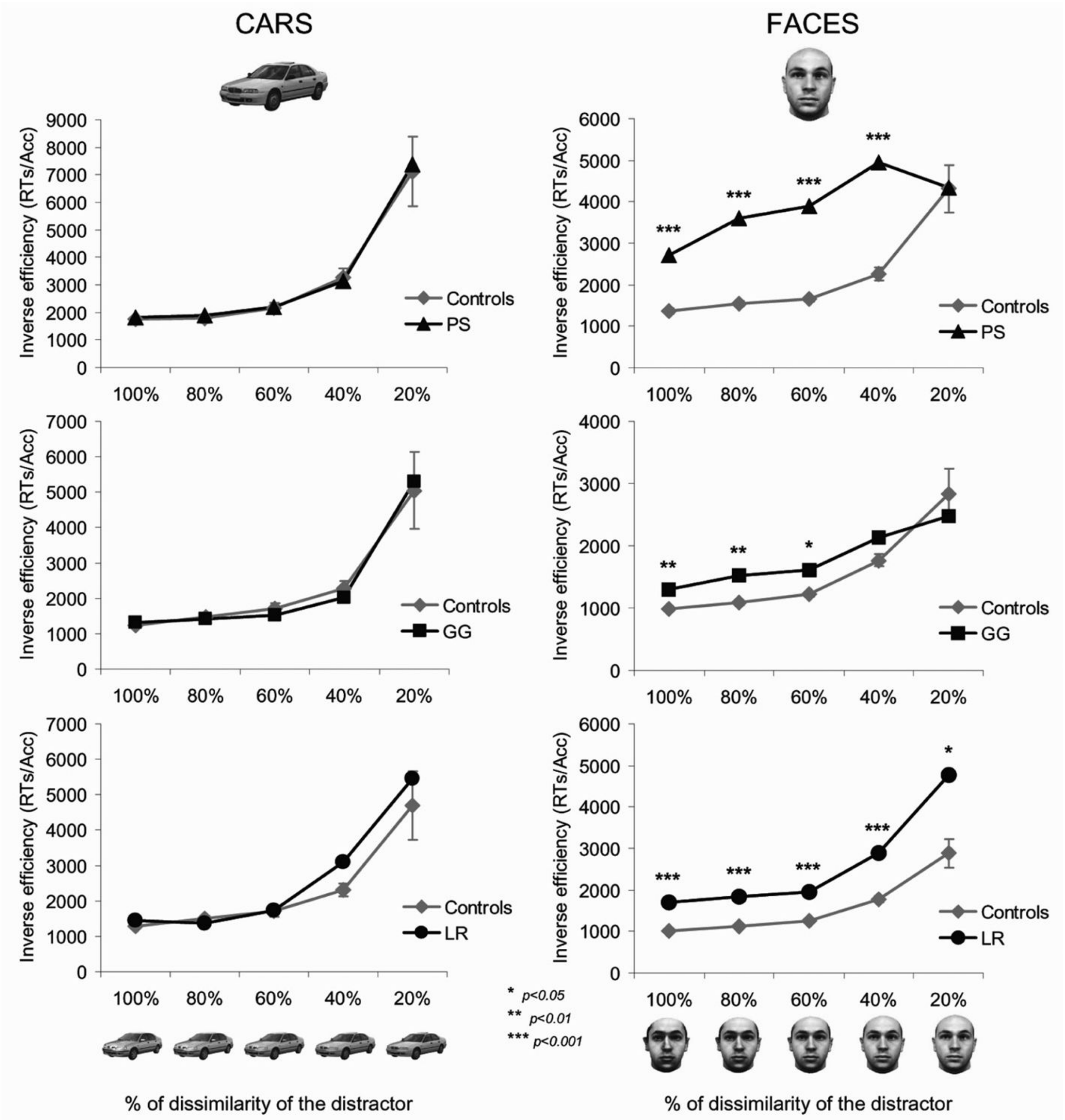

Fig. 1. Results of prosopagnosic patients PS, GG and LR in car and face conditions, according to the level of visual similarity between the target and the distractor.

thermore, there was no evidence of a steeper increase of error rates and RTs with increasing levels of visual similarity, compared to controls. Accuracy and correct RTs were computed in a measurement of inverse efficiency that shows that the three prosopagnosic patients do not differ from controls at any level (see Figure). These data rule out an account of acquired prosopagnosia in terms of a general problem of fine-grained discrimination in a visually homogenous category.

With faces, prosopagnosic patients' performance looked quite different than their own performance with nonface stimuli, and than control participants' performance. At the easiest levels of discrimination, when individual faces differ clearly, the three patients were strongly impaired in accuracy and/or correct response times. Analyses conducted on inverse efficiency measurements showed that GG was impaired for the three first levels: 100% ($t = 3.851$, $p < 0.01$), 80% ($t = 4.272$, $p < 0.01$) and 60% ($t = 2.866$, $p < 0.05$). PS was impaired for the four first levels: 100% ($t = 6.582$, $p < 0.001$), 80% ($t = 10.35$, $p < 0.001$), 60% ($t = 11.26$, $p < 0.001$) and 40% ($t = 6.287$, $p < 0.001$). LR was impaired at each level: 100% ($t = 6.979$, $p < 0.001$), 80% ($t = 5.36$, $p < 0.001$), 60% ($t = 5.134$, $p < 0.001$), 40% ($t = 4.965$, $p < 0.001$) and 20% ($t = 2.012$, $p < 0.05$). However, and most importantly, none of the patient showed a steeper decrease of performance with increasing levels of visual similarity, compared to controls (see Figure).

As expected, the control participants showed significant increases with the degree of similarity between a target and its distractor: the more similar the distractor was to the target the more their performance decreased. This was the case both for car and face stimuli. For photographs of cars, PS, GG and LR showed exactly the same profile of response as the controls. However, for faces, they presented with a quite different profile of performance. They were already well below normal performance at the easiest level of dissimilarity, and their performance slope was no stronger than control participants.

Overall, these results go clearly against the view that acquired prosopagnosia is associated with increasing difficulty at discriminating visually similar items. These observations indicate that the view of prosopagnosia as a more general impairment for fine-grained discrimination in visually homogeneous object categories does not hold.

In conclusion, the fact that there are recognition impairments restricted to faces imply that there are processes that presumably developed through experience to deal efficiently with faces, because these stimuli pose particular challenges for the visual recognition system: faces are indeed highly similar, they are made of multiple (internal) parts, their differences cannot be verbalized easily, we need to individualize them, they undergo fast (expression) and slow (ageing) changes, etc. An ability that would be particularly crucial for face individualization could be configural/holistic processing, that is the ability to process simultaneously facial features and relations between them (see [3,4,8]). This process would be strictly necessary for face recognition, but not for object recognition. Thus, it seems that brain damage in adulthood may lead to selective recognition impairment for faces, perhaps the only category of visual stimuli for which configural/holistic perception is not only potentially at play, but is strictly necessary to individualize members of the category efficiently.

### References

[1] C.M. Bukach, D.N. Bud, I. Gauthier and M.J. Tarr, Perceptual expertise effects are not all or none: Spatially limited perceptual expertise for faces in a case of prosopagnosia, *Journal of Cognitive Neurosciences* **18**(1) (2006), 48–63.

[2] T. Busigny, M. Graf, E. Mayer and B. Rossion, Acquired prosopagnosia as a face-specific disorder: Ruling out the general visual similarity account, *Neuropsychologia* **48** (2010), 2051–2067.

[3] T. Busigny, S. Joubert, O. Felician, M. Ceccaldi and B. Rossion, Holistic perception of the individual face is specific and necessary: evidence from an extensive case study of acquired prosopagnosia, *Neuropsychologia* **48** (2010), 4057–4092.

[4] T. Busigny and B. Rossion, Acquired prosopagnosia abolishes the face inversion effect, *Cortex* **46** (2010), 965–981.

[5] A.R. Damasio, H. Damasio and G.W. Van Hoesen, Prosopagnosia: Anatomic basis and behavioural mechanisms, *Neurology* **32** (1982), 331–341.

[6] C. Faust, Evaluation of posttraumatic organic personality disorders, *Deutsche Medizinische Wochenschrift* **80** (1955), 1237–1239.

[7] I. Gauthier, M. Behrmann and M.J. Tarr, Can face recognition really be dissociated from object recognition? *Journal of Cognitive Neuroscience* **11**(4) (1999), 349–370.

[8] M. Ramon, T. Busigny and B. Rossion, Impaired holistic processing of unfamiliar individual faces in acquired prosopagnosia, *Neuropsychologia* **48**(4) (2010), 933–944.

[9] B. Rossion, R. Caldara, M. Seghier, A.-M. Schuller, F. Lazeyras and E. Mayer, A network of occipito-temporal face-sensitive areas besides the right middle fusiform gyrus is necessary for normal face processing, *Brain* **126** (2003), 2381–2395.
